# Brain enhancement through cognitive training: a new insight from brain connectome

**DOI:** 10.3389/fnsys.2015.00044

**Published:** 2015-04-01

**Authors:** Fumihiko Taya, Yu Sun, Fabio Babiloni, Nitish Thakor, Anastasios Bezerianos

**Affiliations:** ^1^Centre for Life Sciences, Singapore Institute for Neurotechnology (SINAPSE), National University of SingaporeSingapore, Singapore; ^2^Department of Molecular Medicine, University “Sapienza” of RomeRome, Italy; ^3^Department of Electrical and Computer Engineering, National University of SingaporeSingapore, Singapore; ^4^Department of Biomedical Engineering, Johns Hopkins UniversityBaltimore, MD, USA

**Keywords:** cognitive training, brain connectome, electroencephalography (EEG), functional magnetic resonance imaging (fMRI), biomarkers

## Abstract

Owing to the recent advances in neurotechnology and the progress in understanding of brain cognitive functions, improvements of cognitive performance or acceleration of learning process with brain enhancement systems is not out of our reach anymore, on the contrary, it is a tangible target of contemporary research. Although a variety of approaches have been proposed, we will mainly focus on cognitive training interventions, in which learners repeatedly perform cognitive tasks to improve their cognitive abilities. In this review article, we propose that the learning process during the cognitive training can be facilitated by an assistive system monitoring cognitive workloads using electroencephalography (EEG) biomarkers, and the brain connectome approach can provide additional valuable biomarkers for facilitating leaners’ learning processes. For the purpose, we will introduce studies on the cognitive training interventions, EEG biomarkers for cognitive workload, and human brain connectome. As cognitive overload and mental fatigue would reduce or even eliminate gains of cognitive training interventions, a real-time monitoring of cognitive workload can facilitate the learning process by flexibly adjusting difficulty levels of the training task. Moreover, cognitive training interventions should have effects on brain sub-networks, not on a single brain region, and graph theoretical network metrics quantifying topological architecture of the brain network can differentiate with respect to individual cognitive states as well as to different individuals’ cognitive abilities, suggesting that the connectome is a valuable approach for tracking the learning progress. Although only a few studies have exploited the connectome approach for studying alterations of the brain network induced by cognitive training interventions so far, we believe that it would be a useful technique for capturing improvements of cognitive functions.

## Introduction

Recent developments in neuroimaging techniques and related mathematical tools have extended our understanding of neural mechanisms underlying brain cognitive functions. As such, enhancement of cognitive performance or speeding-up learning process through a brain enhancement system is a tangible target of contemporary research. Thus, the enhancement of brain functions has been studied for a wide range of cognitive functions using a variety of techniques (Clark and Parasuraman, 2014).

Such a system for brain enhancement would be beneficial for a wide variety of people and can be based on several techniques. Firstly, patients with neurological disorders (e.g., Alzheimer’s disease, dementia, stroke) or psychiatric disorder (e.g., schizophrenia, major depression, bipolar disorder) would be greatly benefitted if undesirable symptoms can be diminished or rehabilitation can be speeded up by the system (Farah et al., 2004). So far, two enhancement approaches, psychopharmacology and the brain stimulation, have a long history of researches and medical applications. Secondly, healthy elderly people with declined cognitive functions due to aging can be benefited from such a system, as the quality of their daily life would be improved. It is known that aging has detrimental effects on several cognitive functions such as processing speed, working memory (WM) function, executive function, reasoning, and long-term memory (LTM; Park et al., 2002) although some other cognitive functions such as vocabulary (Schaie, 1994) and implicit memory (Fleischman et al., 2004) remain relatively stable or even get improved. Also, substantial evidences have emerged to show that brain can be modified or reorganized throughout the lifespan (Gutchess, 2014). Thirdly, people who are working under extreme circumstances, such as traffic controllers, military personnel, and surveillance system operators, will have a great profit from the brain enhancement system as they need to engage in operations for a long duration with high workloads and pressure, and even a small error in the operations could result in fatal accidents (Pop et al., 2012). Fourthly, struggling students could be benefited from a system that accelerates their learning performance when they are cramming for their examinations, and thus improving their chances for a good job status and salary, which are often dependent on their educational backgrounds (Deary et al., 2007). In fact, it is known that a psychostimulant called methylphenidate (MPH), also known as Ritalin, is sometimes misused by students for boosting cognitive abilities (Talbot, 2009). Finally, even ordinary people can be benefitted from the advantages of such a system, since their quality of life, their reputation in public community, or their performance at workplace could be improved along with the enhancement of the memory function, the attention levels, or emotional states. For the purpose, a variety of interventions such as cognitive training (Klingberg, 2010), neurofeedback (Sulzer et al., 2013), or more directly by brain stimulations, e.g., TMS, tDCS (Hamilton et al., 2011), or psychopharmacological drugs, e.g., MPH, modafini (Repantis et al., 2010) have extensively been studied.

**Figure 1:**
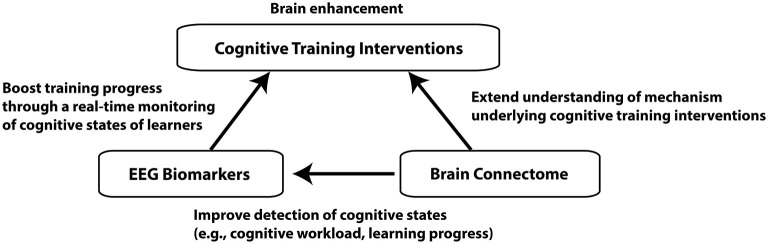
**A schematic diagram depicting connections between the three different topics**. Improvement of cognitive functions through cognitive training interventions is the ultimate goal of the brain enhancement system we propose. EEG biomarkers can facilitate learners’ learning process through a real-time monitoring of cognitive states while brain connectome approach can improve detection of cognitive states as well as understanding of neural mechanism underlying cognitive training.

In this review article, we will mainly focus on the brain enhancement through the cognitive training interventions, in which people perform specific cognitive tasks for improving their cognitive functions (Klingberg, 2010). We will propose that electroencephalography (EEG) biomarkers of cognitive workload can be used for a brain enhancement system to improve the outcome of cognitive training interventions, and the connectome approach can provide further valuable metrics for the assessment of effectiveness of the interventions. For this purpose, three general topics will be covered: cognitive training interventions, EEG biomarkers for cognitive workload, and the brain connectome approach (Figure 1). The purpose of this review article is to bridge between these three different topics. A similar attempt has been made for the combination of brain stimulation and connectome, which will not be covered here (Luft et al., 2014).

Firstly, we will introduce studies on cognitive training interventions, and their effects on the brain activities (Klingberg, 2010). Generally, the cognitive training interventions without any physical or pharmacological interventions would be more desirable for most people because of its relatively low-costs and lower potential risks—undesirable side effects (e.g., headache, dizziness, nervousness, sleep disturbances) can be avoided. Neurofeedback, in which individuals are presented with a feedback signal derived from brain activity that indicates their learning goals, is another technique for brain enhancement which requires no physical interventions and has several common characteristics with the cognitive training. However a significant difference might be the fact that while cognitive training goals include improvements of behavioral performance and accompanying modifications in brain activations, neurofeedback is targeting directly in improving brain activations and consequently increasing cognitive performance.

Secondly, we will introduce EEG biomarkers for cognitive workload, and propose that an adaptive training system using EEG biomarkers based on real-time monitoring of cognitive workload can improve gains of the cognitive training interventions as cognitive overload or mental fatigue during the course of training would reduce or even eliminate the gains of cognitive training (Baldwin and Penaranda, 2012). Owing to recent developments and spreads of neuroimaging techniques such as EEG and MRI, tremendous amounts of studies have been done for investigating associations between mental states and brain activities. Meanwhile, a lot of researchers have developed mathematical methods for revealing biomarkers of brain functions mainly based on advances in signal processing and machine learning techniques (Kothe and Makeig, 2011). The combination of extended knowledge of the mechanism underlying brain cognitive functions and the advanced mathematical techniques would provide more elaborated ways for boosting learning processes during cognitive training.

Finally, we will propose that the brain connectome approach, mainly based on graph theory (Sporns, 2014), would provide further valuable biomarkers for monitoring mental states to accelerate learning process by optimizing cognitive workload during the performance of training tasks. The brain connectome is a relatively new approach for investigating topological architecture of the brain network. Because the brain is a complex network consisting of a number of brain areas dedicated to different functions, it has been suggested that cognitive functions emerged from the dynamic interactions of the distributed areas in large-scale network (Bressler and Menon, 2010). Therefore, brain network analysis would provide further insights into the mechanism underlying the cognitive training, and the graph theoretical network metrics would be useful for discriminating different brain states during the training. We will first provide a general introduction of the network science, and then introduce studies applying the connectome approach to the brain connectivity. Next, we will present our attempts to employ the connectome approach for discriminating different cognitive states as well as relevant studies that demonstrated cognitive state dependent differences in brain networks or changes in brain network evoked by prior experience or cognitive training. Although so far only one study directly examined the changes in network metrics induced by cognitive training interventions (Langer et al., 2013), the studies showing changes of brain connectivity and differences in graph theoretical network metrics would suggest a potential use of the network metrics for the brain enhancement system assisting learners’ learning processes.

## Cognitive Training

### Cognitive Training Interventions

Cognitive training has emerged as a promising alternative to improve cognitive abilities (Lustig et al., 2009; Karbach and Schubert, 2013; Moreau and Conway, 2013). Several studies have been performed to explore the effectiveness of the cognitive training and its effects on neural activities (Klingberg, 2010; Jolles and Crone, 2012). It has also been suggested that even just playing video games could improve perceptual or cognitive abilities (Green and Bavelier, 2003). Because of its ease of use and the numerous potential applications, the cognitive training has attracted substantial public attention, and a lot of computer software for “brain training” are available on web, PCs or smartphones, e.g., Lumosity,^1^ CogniFit,^2^ Cogmed.^3^

In this section, we will introduce neuroimaging studies on cognitive training, which demonstrated changes of brain activations or morphological changes in the brain induced by the cognitive training interventions. Understanding the neural process underlying the cognitive training interventions is of great importance in order to develop the brain enhancement system facilitating learners’ learning processes. The theoretical framework capturing the neural plasticity behind cognitive training is introduced by Hebb, known as Hebbian learning theory (Hebb, 1949). According to this theory, any two neurons or group of neurons that are repeatedly active at the same time they will tend to form stronger associations, and consequently, activity in one will be facilitating activity in the other. Briefly, when neurons fire together, the connection between them is strengthened. This means that when executing a cognitive task repeatedly, the brain areas associated with the cognitive functions engaged in the task will form stronger associations. Hence, we could improve our cognitive abilities through modifications of the brain activations induced by cognitive training interventions.

Major criticism on the cognitive training is about the transferability of training-related performance gain (Lustig et al., 2009). It is likely that performance of the trained task would be improved by the training, but its effects could be limited to the particular trained task (Jaeggi et al., 2008). What most of the people expect of the cognitive training is an improvement of their general cognitive abilities useful in everyday life, not just a better performance specific to the trained task. Therefore, it is of great importance to succeed in reproducing the improved performance gained from training in one task, on another, different task with no prior training on the second (Karbach and Schubert, 2013). Improved performance on untrained, but directly related tasks to the trained task is called “near transfer”, while improvements on untrained tasks which are related, but not directly related to the cognitive abilities is called “far transfer”. In fact, several studies have shown the possibility of such far transfer of practice effect beyond task-specific performance (Klingberg et al., 2002), although its generality remains controversial (Colom et al., 2010). The basic theory behind the transfer is also simple. If a brain sub-network that is engaged in a trained task overlaps with networks related to untrained tasks, these networks will be also strengthened following the Hebbian learning rule, and consequently result in improved cognitive performance on the untrained tasks.

In search of training schemes that can induce transfer effects, WM training has been studied intensively. It is believed that the WM is essential for a variety of higher cognitive functions such as reasoning, problem solving, and decision making (Klingberg, 2010), and moreover, it is considered to be the basis for the general intelligence (Conway et al., 2003). Additionally, WM capacity is crucial for knowledge and skill acquisition, and is closely related to academic achievements and educational success, engaging intense interest from a broad range of people. For this reason, many programs for cognitive training including commercial products such as Cogmed WM Training^4^ or Jungle Memory Program^5^ (Shipstead et al., 2012), are designed to target on the WM capacity. Some studies have demonstrated that the gain of WM oriented cognitive training can be transferred to cognitive control mechanisms (Klingberg et al., 2002), the WM updating process (Dahlin et al., 2008), reading comprehension (Chein and Morrison, 2010), and even to measures of fluid intelligence, a cognitive ability of abstract thinking and adaptation to novel problems (Jaeggi et al., 2008). The fluid intelligence is known to be closely related to professional and educational success (Neisser et al., 1996). Although it is believed that the fluid intelligence is unsusceptible to influences of education, Halford et al. proposed a hypothesis that the WM and reasoning share a common mechanism, providing a framework for improvements of the general intelligence through WM oriented cognitive training (Halford et al., 2007). To verify the hypothesis, Jaeggi et al. trained subjects for 8–19 days with an adaptive dual *n*-back task, in which subjects were required to update the information about spatial locations of visual stimuli and auditory information concurrently, and found the improvements of the fluid intelligence measured by Raven’s Advanced Progressive Matrices test and the Bochumer Matrizen-Test (Jaeggi et al., 2008). Stephenson et al. also found improved scores in two out of four tests for the fluid intelligence by the dual *n*-back task training (Stephenson and Halpern, 2013). Despite a number of successful observations of transfer effects of WM training gain, neural mechanism underlying the WM training interventions remains elusive. Some studies have suggested that the improvements of fluid intelligence could be achieved through cognitive training other than WM training. Colom et al. found similar improvements in two out of four scores measuring fluid intelligence induced by simple speed tasks (Colom et al., 2010). Also the improvement of fluid intelligence was observed only for participants who underwent cognitive training with visuospatial components, and even a visuospatial short-term memory (STM) training improved the fluid intelligence (Stephenson and Halpern, 2013). One promising account for the effects of WM training is that participants’ short-term storage capacity, which is a common factor among STM, WM, executive function, attention, and general fluid intelligence, is expanded through intensive performance of the cognitive training (Colom et al., 2013).

### Effects of Cognitive Training on Brain Activations

In order to develop the brain enhancement system which facilitates cognitive training processes, it is also of great importance to investigate the effects of cognitive training on the brain and to understand what is actually accomplished by the cognitive training. Modulation of brain activation has been demonstrated with a variety of cognitive training interventions, such as the WM training (Hempel et al., 2004; Olesen et al., 2004; Jolles et al., 2010), an attentional training (Mozolic et al., 2010), dual tasks (Erickson et al., 2007), video games training (Maclin et al., 2011), and even meditation training (Tang and Posner, 2014).

A number of studies using functional magnetic resonance imaging (fMRI) have shown that besides improving behavioral performance, intense cognitive training resulted also in changes of the brain activations that were related to the cognitive functions implicated in the tasks used as cognitive interventions were changed by intense cognitive training, accompanying with improvements of behavioral performance (Hempel et al., 2004). Olesen et al. found an increase of brain activity in the areas related to the WM induced by 5 weeks practice of WM tasks (Olesen et al., 2004) while Hempel et al. showed increased activations after 2 weeks of training on a WM task and decreased activations after 4 weeks, suggesting two distinct mechanisms mediating the training effects: an enhancement mechanism for WM and a suppressive mechanism related to automation of processing (Hempel et al., 2004). Furthermore, a training of multi-task processing revealed training-induced reductions in activity of brain areas responsible for stimulus-response associations, attentional control, and response selection process as well as an increase of activity in a region related to executive control (Erickson et al., 2007). Such reductions in brain activity induced by training may reflect increased task selectivity within the areas (Dux et al., 2009). Even thirty hours of training on a video game can induce reduction of activation in attentional control areas, suggesting a reduction of attentional demands after the training (Lee et al., 2012). Interestingly, a WM training has also induced less deactivation in “default-mode network”, which is usually deactivated during cognitive tasks, suggesting more automatic processing after practice (Jolles et al., 2010).

In addition to the changes of activation at brain regions specific to trained tasks, several neuroimaging studies demonstrated transfer effects of cognitive interventions (Dahlin et al., 2008). Dahlin et al. examined whether transfer effect was induced by training on a task that involved “updating”, which is a basic executive function relating to intelligence, WM, and manipulation of information (Dahlin et al., 2008). After 5 weeks of training on a letter memory task, young subjects showed improved performance on 3-back task, but no improvement in the Stroop task. A comparison between pre- and post-changes in the fMRI data that were collected during the training task showed increased activity in the left striatum and decreased activity in fronto-parietal network. As far as the transfer task is concerned, training-induced increases in brain activations were found in the left striatum and the frontal cortex for the 3-back task, but no significant changes were detected for the Stroop task. Additionally, a conjunction analysis revealed that overlap region exclusive to letter memory and 3-back was the left striatum, which is associated with updating, suggesting that the activation of the overlapping brain region during the training induced the transfer to the untrained 3-back task. No transfer was observed in older adults who showed no significant activation in the striatum during the letter memory task. Transfer effect was also examined for affective cognitive control (Schweizer et al., 2013). They found that twenty days of a dual *n*-back task with emotional stimuli (eWM task) induced improved emotional regulation and increased activations in the fronto-parietal demand network, including the dorsal and subgenual anterior cingulate (sgACC), on another task that required emotional regulation. Furthermore, several studies have examined cross-modal transfer effects of WM training (Schneiders et al., 2011, 2012; Buschkuehl et al., 2014). Buschkuel et al. have examined cross-modal transfer effects of 7 days of training of a visuospatial *n*-back task to an auditory *n*-back task, and investigated longitudinal changes of brain activities using perfusion (arterial spin labeling; ASL) (Buschkuehl et al., 2014). They found a transfer effects across modalities, and observed increased perfusion in right superior frontal gyrus, which is thought to be involved in executive control and WM processing. On the contrary, Schneiders et al. failed to observe cross-modal transfer effects for a visual and an auditory WM tasks (Schneiders et al., 2011, 2012). They found a modality-specific training effect for the visual WM training in the right middle frontal gyrus, which is to some extent specific to the maintenance of visual objects in WM, (Schneiders et al., 2011), and that for the auditory WM training in the right inferior frontal gyrus responsible for maintaining auditory information (Schneiders et al., 2012). However, no across-modal transfer effects were detected. One possible account for this discrepancy is that WM training would once increase brain activations in areas associated with executive control, which is shared between WM tasks with different modalities, but further training would decrease the brain activation along with a decrease in cognitive efforts necessary for the performance of the WM tasks (Chein and Schneider, 2005).

Furthermore, such modifications of brain activations induced by cognitive training can also be captured by EEG as well (Maclin et al., 2011). Changes in activtions related to attentional processes triggered by complex game learning were detected in P3 ERP component as well as in δ and α EEG spectral power (Maclin et al., 2011). Moreover, frontal EEG α power during early phase of the game training predicted subsequent learning rates (Mathewson et al., 2012). An improvement of the fluid intelligence induced by WM training and increases in θ and α synchronization have suggested that the WM training has improved not only WM maintenance functions, but also central executive and attentional control (Jaušovec and Jaušovec, 2012). A first-person shooter (FPS) video game enhanced neural processes that support spatial selective attention, as it was shown by increased amplitudes of the later visual ERPs in high-performing FPS players (Wu et al., 2012). Improvements of visual attention allocation, executive attention, and updating function in WM representation has been indicated by increases in ERP components (N160, P200 and P300) after training on a WM task that engaged updating function (Zhao et al., 2013). Modifications of EEG signals induced by training on a game involving dual tasks or a WM task along with behavioral improvements were also observed for elderly adults (Anguera et al., 2013) and dysphoric participants (Owens et al., 2013). Moreover, a meditation training such as integrative body-mind training (IBMT) improved attention, mood, and stress regulation, while it increased frontal midline θ power, where the anterior cingulate cortex (ACC) is suggested to be the generator of the activity (Tang et al., 2009). Thus, several cognitive training programs have shown alterations in brain activities as well as their effectiveness in improvements of cognitive performance. In Table 1, we provide a summary of existing neuroimaging studies showing changes in brain activations induced by cognitive training interventions.

**Table 1:** **Studies showing changes in brain activations induced by cognitive interventions**.

Study	Modality	Training task	Control group	Population	Training period
McKendrick et al. (2014)	NIRS	a dual verbal and spatial WM task	a yoked condition group	YA	5 days
Heinzel et al. (2014)	fMRI	an adaptive *n*-back task	-	OA and YA	4 weeks
Buschkuehl et al. (2014)	ASL	adaptive visuaospatial *n*-back	vocabulary and general knowledge questions	YA	7 days
Zhao et al. (2013)	EEG	Three memory tasks	no training	YA	21–23 days
Schweizer et al. (2013)	fMRI	adaptive emotional dual *n*-back	a feature match training	YA	20 days
Owens et al. (2013)	EEG	an online dual *n*-back task	a nonadaptive dual 1-back task	YA (dysphoric)	2 weeks
Anguera et al. (2013)	EEG	NeuroRacer (a dual task)	single task and no-contact control	OA	4 weeks
Wu et al. (2012)	EEG	a FPS video game	nonaction game control group	YA	10 h
Schneiders et al. (2012)	fMRI	adaptive auditory *n*-back	-	YA	2 weeks
Prakash et al. (2012)	fMRI	Space Fortress vidoegame	only limited game experience	YA	30 h
Mathewson et al. (2012)	EEG	Space Fortress vidoegame	-	YA	20 h
Lee et al. (2012)	fMRI	Space Fortress videogame	only limited game experience	YA	30 h
Jaušovec and Jaušovec (2012)	EEG, NIRS	Five different WM tasks	communication and social skills	YA	30 h
Schneiders et al. (2011)	fMRI	adaptive visual or auditory *n*-back	no training	YA	2 weeks
Maclin et al. (2011)	EEG	Space Fortress vidoegame	-	YA	20 h
Jolles et al. (2010)	fMRI	a verbal WM task	no training	YA	6 weeks
Dux et al. (2009)	fMRI	sensory-motor task (single or dual task trials)	-	YA	2 weeks
Tang et al. (2009)	EEG	a meditation training	a relaxation training	YA	5 days
Dahlin et al. (2008)	fMRI	a letter memory task	no training	OA and YA	5 weeks
Erickson et al. (2007)	fMRI	a dual tasks and a single task	no training	YA	2–3 weeks
Olesen et al. (2004)	fMRI	WM tasks	-	YA	5 weeks
Hempel et al. (2004)	fMRI	*n*-back	-	YA	4 weeks

*Note: EEG: electroencephalography, fMRI: functional magnetic resonance imaging, NIRS: near-infrared spectroscopy, ASL: arterial spin labeling, YA: young adults, OA: old adults*.

### Structural Brain Changes Induced by Cognitive Training Interventions

In addition to the training-induced changes in brain activity, morphological changes can be induced by cognitive practice in the adult brain despite a belief that changes in brain structure are limited to the critical period of development (Draganski and May, 2008). Repeated practice of skills during professional career can induce long-lasting changes in structure of the brain: i.e., London taxi drivers who have substantial experiences to use spatial knowledge for navigation in the complex city were found to have larger gray matter volumes in hippocampus (Maguire et al., 2006), professional typists devoted to the prolonged practice of typing show increased gray matter volume in brain regions related to programming of motor tasks such as supplementary motor area, prefrontal cortex and cerebellum (Cannonieri et al., 2007), violinists and other string players who use the second to the fifth digits of the left hand for fingering the string have larger cortical representation of the digits of the left hand in the primary somatosensory cortex (Elbert et al., 1995).

More directly, several studies have examined the effects of training on the structure of the brain. Modulations of neural structures and functions of the brain can occur for a relatively short period of time as demonstrated by MR-based morphology in conjunction with longitudinal design. Three months of training on juggling task induced a transient expansion of gray matter in the brain areas associated with the processing and storage of complex visual motion for both young and older participants (Draganski et al., 2004; Boyke et al., 2008). Even seven days of the juggling training induced a change in gray matter (Driemeyer et al., 2008) and 6 weeks of the training induced changes in white matter measured with diffusion tensor imaging (DTI) as well as in gray matter density (Scholz et al., 2009). Additionally, real-life intervention such as an intensive preparation for the medical examination, which requires acquisition of substantial amount of new information, could also induce the increment of gray matter in the brain areas known to be involved in memory processes (Draganski et al., 2006). Furthermore, a variety of training tasks other than the juggling training have induced alterations in brain structures. A Morse code training, a sort of language learning, induced a gray matter increase in the left occipitotemporal cortex, which projects to the area involved in language perception (Schmidt-Wilcke et al., 2010). A complex motor skill learning task induced an increase in gray matter volume in the prefrontal cortex, which was positively correlated with performance improvements over time, and a decrease in white matter volume in the prefrontal cortex (Taubert et al., 2010). A memory training induced cortical thickness changes in the right fusiform and lateral orbitofrontal cortex correlated with improvements in memory performance (Engvig et al., 2010). A WM training increased myelination measured by fractional anisotropy (FA) of fiber tracts in the white matter regions adjacent to the intraparietal sulcus and the anterior part of the body of the corpus callosum, both of which are considered to be critical in WM (Takeuchi et al., 2010). A mental calculation training that required WM function induced a decrease in regional gray matter volume in the WM-related regions, which could be attributed to the usage-dependent selective elimination of synapses (Takeuchi et al., 2011). A meditation training increased white matter efficiency in areas surrounding the ACC that is implicated in cognitive control (Tang et al., 2012). Furthermore, a logical reasoning training gain to fluid intelligence was associated with an increase in structural integrity in corpus and genu of the corpus callosum, which connect between homologous cortical areas of the two hemispheres and are considered to be involved in executive functions and WM (Wolf et al., 2014). Taken together, structural brain changes in response to cognitive training interventions were observed for a variety of training schemes, and such changes were mainly found in the brain areas that were supposed to be involved in the training tasks. These observed structural plasticity can be a basis of improvements in cognitive functions through the interventions, suggesting a potential effectiveness of the cognitive training. We provide a brief summary of structural changes in the brain induced by training intervention in Table 2.

**Table 2:** **Studies showing structural brain changes induced by training interventions**.

Study	Modality	Training task	Control group	Population	Training period
Wolf et al. (2014)	DTI	logical reasoning training	-	OA	4 weeks
Tang et al. (2012)	DTI	a meditation training	a relaxation training	YA	4 weeks
Takeuchi et al. (2011)	gray matter volume	mental calculation	placebo, no training	YA	5 days
Takeuchi et al. (2010)	DTI	WM program	-	YA	2 months
Engvig et al. (2010)	cortical thickness	memory training	no training	OA	8 weeks
Taubert et al. (2010)	DTI, gray matter volume	a complex motor skill learning	-	YA	6 weeks
Schmidt-Wilcke et al. (2010)	gray matter density	a Morse code learning	no training	YA	2.5–8 months
Scholz et al. (2009)	DTI, gray matter	juggling	no training	YA	6 weeks
Driemeyer et al. (2008)	gray matter density	juggling	-	YA	7 days
Boyke et al. (2008)	gray matter density	juggling	-	OA	3 months
Draganski et al. (2006)	gray matter density	studying for medical exam	-	YA (medical students)	3 months
Draganski et al. (2004)	gray matter density	juggling	jugglers vs. non-jugglers	YA	3 months

*Notes: DTI: diffusion tensor imaging, YA: young adults, OA: old adults*.

### Summary of Effects of Cognitive Training

In summary, cognitive training can modify activations or the structure of the brain regions directly related to the training tasks along with improvements in behavioral performance of the tasks. As different training tasks can induce changes of brain activations at different brain areas, the selection of training tasks is also an important issue. Although the brain areas engaged by the training tasks can be different dependent on sensory modality, the cognitive tasks recruiting higher cognitive functions, such as WM training or attentional training, are likely to show some transfer effects. Most of the demanding tasks usually recruit higher cognitive functions such as executive function, cognitive control, and attentional control (Buschkuehl et al., 2014). For example, WM training is supposed to induce changes in brain activity in frontal and parietal cortex, both of which are associated with WM capacity (Klingberg, 2010), a WM task that demands emotional regulation evoked increased activation in a part of the ACC, which has been shown to be involved in cognitive control and emotional regulation (Schweizer et al., 2013), an attention training program altered a part of the attentional control system in the prefrontal cortex (Mozolic et al., 2010), sensory motor tasks such as a juggling and a videogame modified cortical regions involved in spatial attention (Prakash et al., 2012) or visual areas specific to motion processing (Draganski and May, 2008), and meditation training program changed brain activities associated with attention, mood, and stress regulation (Tang and Posner, 2014). These results suggest that higher cognitive functions can be improved by cognitive training interventions regardless of sensory modality involved, and the changes in brain activations after cognitive training can be captured by a variety of neuroimaging techniques including fMRI, EEG, fNIRS and structural MRI. Although it is difficult to utilize functional or structural MRI for real-time tracking of training-induced changes of the brain due to their costs and portability, some of the neuroimaging techniques such as EEG can be useful for monitoring alterations in brain activity during the course of cognitive training.

## EEG Biomarkers for Cognitve Workload

In the previous section, we introduced studies showing functional and structural changes of the brain induced by cognitive training interventions. These studies have shown that human cognitive functions could be improved through cognitive interventions if the brain regions implicated in trained tasks overlap between trained and untrained target tasks. In the cognitive training, subjects are required to repeatedly perform behavioral tasks such as WM tasks or video games. As a result of intense involvements of the brain regions during the course of the training, connections among the regions would be enhanced, leading to improved cognitive performance. To engage the brain regions effectively, individualized adaptive training platforms can be useful tools. Also, the neuroimaging techniques including EEG can capture changes in cognitive performance which could be potentially used as biomarkers for the brain enhancement system and facilitate learners’ learning process through tracking learning progress or monitoring mental states.

For example, cognitive overload would induce a reduction of learner’s motivation and mental fatigue, both of which hamper the effectiveness of cognitive training interventions. Thus, the cognitive training can be facilitated using passive Brain-Computer Interface (BCI) system, which utilizes biomarkers derived from the brain signal and adapts to the user’s performance without the purpose of voluntary control of the system (Zander and Kothe, 2011). Through a real-time monitoring of cognitive workload of learners, the system can flexibly be adjusted to avoid overloading learners’ cognitive resources and to keep the learners’ engagement and motivation, speeding up the learning progress (Baldwin and Penaranda, 2012). Additionally, individual differences in learners’ learning rate can be predicted by EEG biomarker (Mathewson et al., 2012), suggesting that combinations with other cognitive training or neurofeedback training which improve the EEG biomarker could optimize training of targeted cognitive functions. In this section, we will first provide a general introduction of EEG biomarkers, which have been studied mainly for BCI and Neurofeedback, and discuss a potential use of biomarkers for increasing effectiveness of cognitive training interventions. As it is practically difficult to use fMRI or structural MRI to monitor learners’ learning process in real-time due to its costs and portability, we will focus on EEG biomarkers here. Then, examples of neurophysiological biomarkers for cognitive workload will be introduced, which can be used for optimizing cognitive workload of a training task to keep learners’ concentration and motivation.

### EEG Biomarkers: BCI and Neurofeedback

The biomarkers based on EEG have been studied extensively for the BCI, which enables users to control computers or devices through the neurophysiological signals, mainly because of ease of use and low cost (Graimann et al., 2010). Mathematical techniques developed for the BCI system can also be employed for the assistive system of cognitive training interventions. In general, the detection of mental states consists of three stages: pre-processing, feature extraction and selection, and classification. The pre-processing can include artifact removals, spatial filtering, and temporal filtering. After the pre-processing, specific properties of the signal will be extracted over window, useful features will be selected for dimensional reduction, and the selected features will be subjected to classification, which accounts for difference in mental states. Classification enables the system to adapt to individual difference in learners, but the selection of feature space to be extracted is critical for detection of cognitive states, and requires understanding of mechanism behind the cognitive states.

In the BCI systems, various types of EEG signals such as modulations of sensorimotor rhythms (SMR) during motor imagery (Pfurtscheller et al., 1997), event-related potentials (Farwell and Donchin, 1988) or slow cortical potentials (SCPs; Birbaumer et al., 1999) are utilized for discriminating mental states. In any case, it is well known that users often have to be trained to control the BCI system properly, suggesting a need of adjustments to individual difference in signals (Neuper and Pfurtscheller, 2010). In the course of the training for the control of BCI, the user is repeatedly presented with feedbacks indicating performance of the system, and needs to learn voluntarily generating specific patterns of brain signal, which is detectable by the BCI system. Thus, a kind of neurofeedback training for the BCI control is necessary. Actually, the only difference between typical BCI systems and neurofeedback training is how to use biomarkers. For the BCI system, detected mental states would be used for controlling devices and the training would be done through improvements of behavioral performance in the control while the neurofeedback training would be achieved through direct modulations of brain signal.

EEG-neurofeedback has been examined with various types of EEG biomarkers, e.g., up- or down-training of the SMR, the β1 ratio, the θ/α ratio, γ, etc., and improved cognitive functions including sustained attention, orienting, executive functions, spatial rotation, procedural memory, recognition memory have been repeatedly reported through the EEG-neurofeedback training (Gruzelier, 2014). The successful outcomes of the neurofeedback training suggest that the mental state revealed by the EEG biomarkers could provide quantitative metrics for guiding learners to obtain certain mental state suitable for efficient learning or performing specific tasks. In the neurofeedback training, the desired brain state would be achieved through associative learning, in which the association between the desired state and reinforcing feedback stimulus revealing the brain activity is learned (Sulzer et al., 2013). For the adaptive system for effective training, such neurofeedback techniques could be used to optimize learners’ mental state to facilitate their learning progress.

### Neurophysiological Biomarkers for Cognitive Workload

The assessment of cognitive workload based on neurophysiological biomarkers, particularly EEG biomarker has been of great interest and been extensively studied (Kothe and Makeig, 2011), and the spectral analysis of the EEG waveforms is a powerful tool for the assessment of the mental states during performance of cognitive tasks (Kohlmorgen et al., 2007).

To search for features of EEG signals associated with task demands, an increase of the EEG power-spectrum in the θ bands (4–7 Hz) at frontal sites and a decrease in the α bands (8–12 Hz) over parietal sites have been investigated (Borghini et al., 2014). The increase of frontal θ activity has been observed for high cognitive demand or high mental effort (Berka et al., 2007), and is considered to reflect attentional process to allocate cognitive resources (Gomarus et al., 2006) while the decrease of α power may reflect semantic LTM processing (Klimesch, 1999).

These kinds of neurophysiological biomarkers can be used for on-line monitoring of mental states. Aricò et al. developed a framework for classification of multiple levels of mental workload during a simulated flight based on EEG and ECG signals (Aricò et al., 2014). In this study, subjects performed the Multi Attribute Task Battery (MATB; Comstock and Arnegard, 1992), which includes a variety of simulated tasks involved in a flight scene, over three different difficulty levels (cruise flight phase, flight level maintaining, and emergencies) during recordings of EEG and ECG signals. The classifiers were trained offline for discriminating cognitive workload, and then tested with the other data where difficulty levels were changed dynamically. The stability of the classifier parameters was tested as well. The workload was assessed based on fusion workload index: a combination of EEG and ECG based workload indices. For the EEG-based workload index, the power spectral density (PSD) was evaluated, and the stepwise linear discrimination analysis (SWLDA) was used to select the most relevant spectral features to discriminate workload levels. For the ECG-based workload index, the PSD was estimated for R-peaks extracted from the ECG signal, and the relevant features were selected using the SWLDA. The derived workload indices were subjected to the SWLDA to calculate the best estimation of coefficients of a linear combination of the EEG and ECG-based workload indices for discriminating the workload levels. The authors demonstrated that the proposed system was able to evaluate multiple levels of mental workload, and the classification parameters were stable within a week.

The frontal θ and parietal α power can be changed along with a change of cognitive efforts induced by training of a task. In the study by Borghini et al., subjects trained on the MATB for 5 days while EEG signals were recorded at first (T1), third (T3) and fifth (T5) day of the training (Borghini et al., 2015). The frontal θ power increased from T1 to T3, and then decreased at T5 and the parietal α power showed an opposite pattern while performances of the MATB task continued to improve during the training. This result seems to reflect changes in attentional demands in the course of the training. We may be able to monitor the progress of cognitive training through such biomarkers associated with allocation of attentional resources.

In summary, there exist several evidences showing that neurophysiological biomarkers can discriminate between different levels of cognitive workload. Although further developments would be necessary for applications based on integration of such biomarkers into an adaptive training system, classification of cognitive workload can be a useful measure for assisting cognitive training and increase its effectiveness by controlling the difficulty levels of the training task. Actually, as mentioned above, some studies have shown changes in frontal θ power after WM training (Jaušovec and Jaušovec, 2012) or training that uses a complex video game (Anguera et al., 2013), suggesting that these EEG biomarkers can be useful for tracking the progress of cognitive training.

## Brain Connectome Approach

The brain connectome is a useful approach not only for understanding brain cognitive functions, but also for extracting biomarkers that could discriminate between different brain states. By using network metrics to represent a feature space for classification, detection of mental states of learners could be enhanced. In this section, we will introduce the network science and its application to brain networks. Firstly, a general introduction of the network science will be provided. Secondly, prior studies using brain connectome approach will be introduced. Then, we will discuss why the connectome approach is beneficial to the brain enhancement system and how it can boost the learning progress. Finally, we will introduce prior studies showing differences or modifications of the brain connectivity patterns, which suggest potential use of brain connectome approach for the brain enhancement system.

### Network Science

It is widely known that brain is a complex network consisting of brain regions dedicated to different kinds of cognitive functions. Furthermore, accumulating evidences support that cognitive functions emerge from the dynamic interactions of distributed brain areas in large-scale networks (Bressler and Menon, 2010). Thus, in order to understand the neural mechanism behind the cognitive training interventions, it would also be important to study alterations in the brain network. The network science, largely based on graph theory, is a useful methodology for investigating an architecture of a complex network and has been employed for studying the brain network (Bullmore and Sporns, 2009; Sporns, 2014). Graph theory is a field of mathematics, aiming at studying topological architecture of networks, and has a long history, dating back to 1736 when a pioneering Swiss mathematician, Leonhard Euler, published the paper on the famous “Seven Bridges of Königsberg” problem. More recently, Watts and Strogatz employed graph theoretical approach to show “small-world” structure of complex networks derived from empirical data (Watts and Strogatz, 1998), leading to the rise of network science as a mathematical tool for studying structure and functions of a wide variety of complex systems from neuroscience, social science, physics, biology, computer science, etc.

In graph theory, networks are represented as “graphs” that consist of objects (“nodes” or “vertices”) and connections or relationships between them (“edges”). The topological properties of complex network are quantified by a wide variety of measures, such as small-worldness, modularity, hierarchy, centrality, and the distribution of network hubs (Bullmore and Sporns, 2009). For example, the degree of a node is the number of connections linking to the other nodes, and the probability distribution of the node degree over the whole network is called the degree distribution: a useful measure for investigating a global architecture of a network. Clustering coefficients quantify the degree of mutual connections between the nearest neighbors of a node. High clustering leads to high efficiency of local interactions and robustness. Shortest path length is the minimum number of edges necessary for a node to reach to another. The inverse of the path length can be used to quantify global efficiency of information transfer of the network. Connection density is a proportion of actual connections to the total possible connections. High connection density indicates high physical cost of a network. The centrality of a node represents its importance in communication, and several measures for node centrality has been proposed, such as degree centrality, eigenvector centrality, closeness centrality, betweennes centrality, and so on. For example, the degree centrality is the simplest centrality measure: the degree of a node, while Betweenness centrality is measured by counting the number of paths between the other nodes passing through the node for taking the shortest route. Nodes with high centrality are called hubs, and can be used for assessing robustness of the network by deleting them. Complex networks often consist of a number of modules composed of locally interconnected nodes with few connections to those in different modules. Hubs can have different roles in this complex network architecture. Provincial hubs are connected mainly to nodes inside their own modules while connector hubs have connections with nodes in other modules. Further details about the formulation of these network metrics and their interpretation can be found in recent reviews of this topic (e.g., Rubinov and Sporns, 2010).

Graph theoretical network analysis allows us to quantitatively study topology of networks. A number of studies utilizing graph theoretical measures have revealed that most real world networks including brain network had non-trivial topological features. Watts and Storogatz have shown that a variety of networks have “small-world” properties, based on empirical examples including social network of film actors, power grid, and the neural network of the worm Caenorhabditis elegans (C. elegans) (Watts and Strogatz, 1998). The “small-world” network, by analogy with the small-world phenomenon (popularly known as six degrees in separation), has topological properties somewhere between two extreme cases, i.e., regular and random networks. The small-world network can be characterized by two independent graph theoretical measures introduced above, namely the clustering coefficients and average shortest path length. Regular network is highly clustered, but has a large path length: it is robust, yet inefficient in information transfer. Random network, on the other hand, has a small path length along with a small clustering coefficient, indicating that it is efficient, but is not robust. Small-world network is the intermediate between the regular and the random network, and consists of a number of clusters or modules, interlinking with each other via hubs. Such modular architecture of the small-world network enables global efficiency of information transfer and robustness to perturbations at the same time. It is believed that the small-world architecture is a fundamental principle of a diversity of complex networks, e.g., social, economical, biological, and neurological networks.

Scale-free network, whose degree distribution follows a power law, is also an important concept in the network science (Barabási, 2013). The scale-free property of the network means that some “hub” nodes have a large number of connections while a majority of nodes have only a few connections, which is considered to enable rapid information transfer, minimal wiring costs, and balancing between local and global communications. Such scale-free property has also been found in a variety of networks in the natural world.

### Brain Connectome Approach to Large-Scale Human Brain Network

A number of studies have attempted to employ graph theoretical approaches for the brain network analysis (Sporns, 2014). For large-scale human brain networks, the nodes are usually considered to be brain regions or sensors (e.g., voxels for fMRI and electrodes for EEG/magnetoencephalography (MEG)) while the edges can be derived from different, but relevant forms of connectivity, i.e., anatomical connectivity (AC), functional connectivity (FC), or effective connectivity (EC). AC, also called structural connectivity, is the axonal-fibers (white matter) pathways usually acquired by DTI or diffusion spectrum imaging (DSI). FC is defined as temporal dependency between activities of distributed and often spatially distant brain regions without explicit reference to causal effects, normally monitored via fMRI. EC represents causal interactions between brain regions, defined as an influence of one system on another. The causality can be inferred through network perturbations, or the temporal ordering of events. As estimations of EC usually require high temporal resolution, signals from EEG, electrocorticogram (ECoG) or MEG are used. The distinction between FC and EC in neuroimaging studies is important when considering several aspects of functional organization (Friston, 1994). Once the connectivity pattern is provided, regardless of which modality is employed for deriving the network, graph theoretical approaches can be applied for investigating its network architecture. The functional and EC derived from functional data can dynamically change even at rest and do not necessarily match with the AC and should not be interpreted as it is (Honey et al., 2009; Hermundstad et al., 2013).

To obtain connectivity patterns of the large-scale network from brain activity, a variety of techniques have been proposed. For FC, correlation analysis can be used regardless of the modality of signals while several techniques have been proposed for EC, depending on modality of signals. To derive EC from electrophysiological signals such as EEG and ECoG, the Granger causality analysis based on multivariate autoregressive model (MVAR) can be used to determine the directional interaction among electrophysiological signals (Astolfi et al., 2007). The directed transfer function (DTF; Kamiński and Blinowska, 1991) and the partial directed coherence (PDC; Baccalá and Sameshima, 2001) have been used to estimate such causal relationships. To derive EC from fMRI data, Granger Causality Modeling (GCM) and Dynamic Causal Modeling (DCM) have been proposed (Valdes-Sosa et al., 2011). Approximately, GCM is data-driven while DCM is hypothesis-driven. Once connectivity maps are obtained regardless of techniques used for deriving connectivity patterns, graph theoretical approaches can be employed to investigate the network properties.

Graph theoretical network metrics demonstrate topological architecture of the brain network, such as global or local efficiency of information transfer, small-worldness, and a modular structure of the network. Studies employing graph theoretical metrics on human large-scale structural brain network have exhibited robust small-world properties, i.e., high clustering coefficients with relatively small mean path length, for structural brain networks derived from diffusion MRI (Hagmann et al., 2007) as well as those from cortical thickness (He et al., 2007). Similarly, the small-world properties have also been shown for human brain functional networks based on neurophysiological data, such as a task fMRI (Eguíluz et al., 2005; Kinnison et al., 2012; Breckel et al., 2013), resting-state fMRI (Salvador et al., 2005; Achard et al., 2006; Achard and Bullmore, 2007; van den Heuvel et al., 2008), EEG (Micheloyannis et al., 2006, 2009; Stam et al., 2007; Smit et al., 2008; Boersma et al., 2011; Langer et al., 2012; Sun et al., 2014a), and MEG (Stam, 2004; Deuker et al., 2009). Also, the scale-free organization has been reported for the functional network derived from fMRI (Eguíluz et al., 2005; Achard et al., 2006; van den Heuvel et al., 2008) and EEG (Lee et al., 2010). These findings consistent among structural and functional brain network reveal that the small-world and scale-free property are fundamental principles of the brain networks, suggesting that the brain networks have evolved to achieve high efficiency of information transfer between nodes at low connection cost with robustness to perturbations.

Furthermore, several studies have shown that the human brain network has some “hub” regions that work as core regions linking between brain regions (van den Heuvel and Sporns, 2013b). The hub regions were identified for both structural brain networks (He et al., 2007; Hagmann et al., 2008; Iturria-Medina et al., 2008; Gong et al., 2009; van den Heuvel and Sporns, 2011, 2013a; Nijhuis et al., 2013) and functional brain networks (Achard et al., 2006; Cole et al., 2010b; Tomasi and Volkow, 2011a,b; Zuo et al., 2012). The identified hub areas were relatively consistent among the studies regardless of the modality used for obtaining the brain networks, and mostly included parietal and prefrontal regions, such as precuneus, anterior and posterior cingulate gyrus, and the superior frontal gyrus (Bullmore and Sporns, 2009; van den Heuvel and Sporns, 2013b). These hub regions are believed to be responsible for multimodal or integrative function. In fact, the precuneus is a part of the default mode network, and has been suggested to be involved in visuospatial imagery, episodic memory retrieval, self-processing, and consciousness (Cavanna and Trimble, 2006), while the superior frontal gyrus contributes to various cognitive functions, such as WM and attention (Petrides, 2005). The damage to these regions could result in drastic changes of stability and efficiency of the network (Sporns and Zwi, 2004). Such hub regions, in particular the fronto-parietal brain network (FPN), may work as a flexible hub, which implement cognitive control by biasing information flow across sub-networks depending on task demands (Cole et al., 2013).

As revealed by the graph theoretical approach, the brain has a modular architecture with small-world network attributes. Each module, probably corresponding to anatomically or functionally defined brain regions, has dense connections within the module for local processing of information, and is implicated in a particular function, exhibiting functional segregation of local areas. On the other hand, the modules are interconnected with each other through short- or long-range connections through hubs, implying functional integration of globally distributed brain areas. In fact, human brain structural network derived from co-variation of regional gray matter volumes measured using MRI and DSI exhibited hierarchical modular architecture with 2–3 levels (Bassett et al., 2010). It is likely that such hierarchical small-world network architecture enables the coexistence of functional segregation and functional integration within a single brain network. Also, these findings are consistent with those for the resting-state FC, which have shown the existence of a number of sub-networks consisting of functionally linked brain regions (Cole et al., 2010a). Therefore, the brain has an inhomogeneous architecture, and each brain region has different functions with different degree of importance. As such, brain regions dedicated to the relevant cognitive functions are engaged depending on cognitive demands, and work together as a network to communicate and influence one-another to produce coherent experiences and behavior. Such sub-networks could be divided into “intrinsic” and “evoked” functional network architecture, where the intrinsic network serves as a standard state of the brain and relatively small changes of the task-evoked networks support task-specific demands (Cole et al., 2014).

In summary, functional and structural connectivity patterns of the human large-scale brain network can be obtained by means of a variety of neuroimaging and mathematical techniques. A number of studies have been conducted to investigate the topological architecture of the brain network using graph theoretical network metrics, and have shown that the human brain network had small-world network characteristics, scale-free organization, and a modular structure, suggesting applicability of network analysis for elucidating neural underpinnings of human cognitive functions.

For the brain enhancement through cognitive training, elucidation of the brain network architecture is of great importance for several reasons. Firstly, cognitive training can affect sub-network consisting of spatially distributed, but functionally relevant brain regions even when improvement of activation in a single brain region is targeted. Secondly, a degree of impact of cognitive intervention can differ among brain regions, depending on its topological property of the brain network. To be specific, hub regions that link with other sub-network can have greater influence compared to other brain areas. Thirdly, the graph theoretical network metrics can simply be additional biomarkers for monitoring mental states of learners during the training, enabling the adaptive learning system depending on the learner’s mental state to facilitate the learning progress. Finally, although reduced activation in brain regions responsible for attentional control and mental efforts has been observed after the cognitive training and is considered to indicate relative automaticity of behavior induced by the training (Prakash et al., 2012), it cannot be accounted for in terms of the improved “efficiency” of neural function (Poldrack, 2015). The connectome approach can provide another perspective for the effects of the cognitive training in terms of changes in cost of information transfer within the network. Additionally, FC strength was spatially correlated with regional cerebral blood flow (rCBF), particularly in the default mode network and executive control network, and the coupling between blood supply and FC in the lateral-parietal lobe was modulated with task demands (Liang et al., 2013), suggesting that FC analysis would provide effective tools for measuring changes of energy consumption induced by cognitive training interventions. Therefore, it is getting more important to consider about modulations of the brain network architecture induced by the cognitive training in order to develop the assistance system for the cognitive training.

We have introduced graph theory as a tool for investigating the topological architecture of complex networks, and its applications to the human large-scale brain network. In the studies introduced above, the graph theoretical network metrics have been used to show prevailing attributes of the brain network, such as small-world architecture, scale-free organization, or hub brain regions. In the next section, we will discuss its applicability for demonstrating difference in the brain functional network architecture, which could discriminate cognitive states.

### Difference in the Brain Functional Network

**Figure 2:**
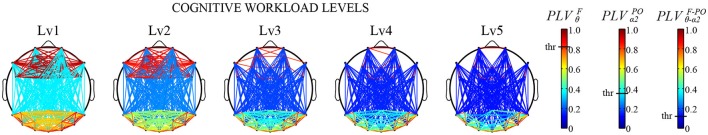
**Cross-frequency causal interactions revealed by Phase Locking Values (PLV) for multiple cognitive workload levels during a mental arithmetic task**. Three different thresholds have been applied to each type of coupling (F/θ, POα2, CFC) (Adapted from Dimitriadis et al., 2014). a permission will be obtained from the publisher after acceptance, the image will be replaced with high-resolution version.

The FC patterns of the brain network can be different depending on cognitive states or states of mind, suggesting that functional brain network can be used as biomarkers for detecting mental states. For example, the functional interaction between cell assemblies revealed by the human EEG coherence, which is the correlation coefficient in the frequency domain, was higher during memory encoding phase for subsequently recalled words compared to forgotten words (Weiss and Rappelsberger, 2000). In one study, Astolfi et al. showed different cortical connectivity pattern during observations of TV commercials between subsequently remembered and forgotten ones (Astolfi et al., 2008). They also demonstrated that the parietal areas received a larger amount of the incoming flow of information during the observation of TV commercials that were remembered than that of forgotten ones. Even more precise differences in cognitive states can be predicted by connectivity patterns of the brain network. Through calculating the cross-frequency causal interactions (Canolty and Knight, 2010) between frontal and parieto-occipital sites, Dimitriadis et al. estimated effective networks derived from EEG signals during a mental arithmetic task with different cognitive workload levels (Dimitriadis et al., 2014). The tensor subspace analysis (TSA) based learning was then used to extract features that can discriminate different cognitive workload levels based on FC patterns. These features achieved a remarkable high correct-recognition-rate (96%) for classification of the task difficulties, suggesting that the FC patterns based on cross-frequency couplings between subregions can become powerful biomarkers for measuring cognitive workload levels (Figure 2). Also, multivariate pattern analysis (MVPA) on EEG FC patterns demonstrated successful classification of mental fatigue states from vigilant state at an accuracy of 81.5% (Sun et al., 2014b). These results have suggested that a combination of FC patterns and advanced mathematical tools could provide powerful biomarkers of cognitive states.

**Figure 3:**
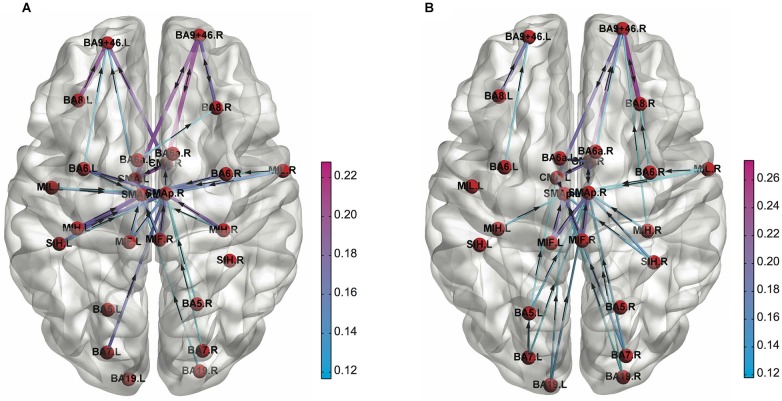
**Functional connectivity patterns in the low alpha (8–10 Hz) frequency band obtained for (A) 1st and (B) 4th quartile during the PVT task performance**. The cortical connections are weaker at left prefrontal cortex compared to right one in the 4th quartile (Adapted from Sun et al., 2014a).

In addition to the FC pattern itself, differences in the topological architecture of the brain functional network can be quantified in terms of graph theoretical network metrics. Kitzbichler et al. have found that the brain functional network derived from human MEG data became more globally efficient, less clustered, and less modular network configuration as cognitive efforts got greater during a WM task (Kitzbichler et al., 2011). Furthermore, Sun et al. employed the graph theoretical metrics on FC patterns derived from lower α band (8–10 Hz) of EEG data to investigate small-world network properties due to a decline in vigilance caused by performing an attention demanding task (Psychomotor Vigilance Test; PVT; Sun et al., 2014a). They found a decrease in efficiency of global information transfer revealed by increased weighted characteristic path length, and an asymmetrical pattern of connectivity (right > left) in fronto-parietal regions due to mental fatigue (Figure 3). Such difference in graph theoretical network metrics was found between rest and a task performance for the functional network derived from MEG (Bassett et al., 2006) and fMRI (Cao et al., 2014; Taya et al., 2014). Also, the graph theoretical metrics on fMRI functional network could discriminate between resting-state and sensory stimulation (Moussa et al., 2011), different cognitive load during a WM task (Ginestet and Simmons, 2011), different cognitive states in an emotional and a motivational task (Kinnison et al., 2012), and between an intentional and an incidental learning of words during neuropsychological tests (Kuhnert et al., 2013). The small-worldness of EEG functional network was different between during rest and mathematical thinking (Micheloyannis et al., 2009) and some network metrics of a combined EEG and MEG functional network was different between low- and high-memory load during a visual WM retention period (Palva et al., 2010). Furthermore, graph theoretical network metrics or network modularity of fMRI functional network were different depending on sleep levels or conscious levels (Ferri et al., 2007; Spoormaker et al., 2010; Tagliazucchi et al., 2013; Uehara et al., 2014).

The graph theoretical network metrics can be effective in discriminating individual differences in mental or cognitive abilities as well. Van den Heuvel et al. have demonstrated a strong negative association between the normalized characteristic path length of the intrinsic brain network derived from resting-state fMRI data and intelligence quotient (IQ), suggesting that human intellectual performance is related to how efficiently the brain integrates information between brain regions (van den Heuvel et al., 2009). Moreover Langer et al. have shown that clustering coefficient and path length of the functional network derived from resting EEG were strongly related to general intelligence evaluated by Ravens advanced progressive matrices: higher small-worldness for higher general intelligence (Langer et al., 2012). Although the network metrics can also be useful biomarkers for diagnosis of mental disorders (Bassett and Bullmore, 2009), we will not discuss about this case here.

In summary, the brain functional network can show different connectivity patterns depending on cognitive states such as cognitive workloads or sleep stages, and such difference can be extracted and be classified using advanced mathematical techniques. Furthermore, the graph theoretical metrics that quantify topological architecture of complex networks can also discriminate between cognitive states and individual differences in intelligence. These results have suggested that graph theoretical network metrics on functional network can be useful biomarkers for monitoring and optimizing cognitive states of learners during cognitive training.

### Modulations of the Brain Network Induced by Cognitive Training Interventions

We have introduced a number of examples showing that the brain FC patterns could be different depending on cognitive states or even individual cognitive abilities. For assisting the cognitive training, it is important to know whether the brain functional network could be changed through cognitive interventions. Although only one study has directly examined the effect of cognitive training interventions on topological architecture of the brain functional network so far (Langer et al., 2013), a number of studies have examined the effect of cognitive interventions on the brain network in different time scales, and found the modulation of the brain network induced by prior experiences including the cognitive training, as introduced below.

There are a number of studies showing modifications of the brain FC induced by cognitive or perceptual task performance. Albert et al. (2009) have shown that a motor learning could modulate subsequent neural activity during rest and the changes in resting activity were not limited to immediately after the learning, but persisted after 4 min of unrelated task. Stevens et al. (2010) have examined the effects of preceding exposure of visual stimuli from distinct categories (i.e., faces or scenes) on FC between frontal networks and category-preferential visual regions during rest. They found that increased couplings between a frontal brain region (rIFG) and category-preferential visual regions after each task, and subsequent memory performance was predicted by the degree of modulation of the couplings. These findings have indicated that resting-state FC could be modulated by perceptual or cognitive tasks at least in a short-term (minutes).

Such modulations of resting-state connectivity induced by prior experience can last for longer duration (days or weeks). Lewis et al. (2009) have investigated the effects of intense training (2–9 days) on a visual perceptual task on resting-state FC, and found that the FC between trained visual cortex and dorsal attention regions became more negatively correlated while that between untrained visual cortex and several default regions became less negatively correlated after learning. Changes of resting-state FC were found for WM training as well. Six weeks of a verbal WM training induced modifications in resting-state FC and the change of frontoparietal connectivity was positively related to performance improvement while that of default network connectivity was negatively correlated (Jolles et al., 2013). Also, 27 days of the WM training program increased FC between mPFC and precuneus and that between mPFC and right posterior parietal cortex and right lPFC (Takeuchi et al., 2013). Even some weeks of training of video games changed FC during game playing (Voss et al., 2012) and rest (Strenziok et al., 2014).

The changes in brain network induced by cognitive training interventions can be observed in structural connectivity as well. Takeuchi et al. have demonstrated that 2 months of WM training increased FA in the white matter regions, possibly attributable to increased myelination after training (Takeuchi et al., 2010). Additionally, Wolf et al. investigated the transfer capability of general fluid intelligence related cognitive training in elderly subjects (Wolf et al., 2014). They examined structural integrity measured by DTI immediately after a 4-week training and a 3-month follow-up period. Although only 22% of subjects showed successful long-term transfer effects, transfer of training gains was associated with a higher degree of structural integrity.

In addition to the individual connectivity between brain regions, the graph theoretical approaches can be employed to examine changes in global characteristics of the brain network. Bassett et al. investigated reconfiguration of modular structure of the FC induced by 3 days of a motor learning task (Bassett et al., 2011). They found that some of the brain regions showed consistent community allegiance (low-flexibility nodes) while other regions constantly shift allegiance (high-flexibility nodes). Additionally, the network flexibility first increased and then decreased during the learning process. The authors have also examined changes of modular structure over thirty-day of a motor skill training, and demonstrated that the separation between core and periphery nodes decreased over the course of training, and good learners tend to have greater separation than poor learners (Bassett et al., 2013). Even 4 days of learning of complex hand coordination pattern induced changes in topological architecture of the fMRI functional network (Heitger et al., 2012). Additionally, Breckel et al. have demonstrated prolonged changes in the global architecture of the resting-state brain network during a series of task performance (Breckel et al., 2013). They compared topological properties of the functional brain network derived from fMRI data before a sustained attentional task performance and those after the task, and showed that the post-task functional network had more clustering, less global efficiency, and less long-distance connections, suggesting a reduction in network integration due to the task performance. These changes in network architecture were still observed after 6 min of resting state. Interestingly, such changes in information transfer efficiency of the brain functional network were induced by a cognitive training intervention (Langer et al., 2013). Langer et al. investigated effects of intensive WM training on the EEG functional network. They first confirmed that WM performance was correlated with power in the θ frequency band, and the WM training increased the θ power. Then, they found the global efficiency of the functional network in the θ band was correlated with higher WM performance before training, and WM training induced increase of small-world topology revealed by an increase of the clustering coefficient and a decrease of the path length in the majority of the subjects.

Taken together, prior sensory or cognitive experience and cognitive training interventions can have even prolonged effects on the brain structural and FC. In addition, motor training and WM training induced changes in topological architecture of the brain network. Although only one study has directly examined the effect of cognitive training interventions using brain connectome approach so far (Langer et al., 2013), alterations in the brain connectivity patterns can also be captured by graph theoretical network metrics such as clustering coefficients or path length. Such changes in topological architecture and information transfer efficiency of the brain functional network can be attributed to improvements in cognitive processing related to the trained tasks, enhancements of general intelligence, changes in attentional levels, difference in cognitive workload, or reconfiguration of brain sub-networks involved. These results have suggested that the network metrics can be useful biomarkers, not only for monitoring cognitive states, but also for tracking effects of the cognitive training interventions. A summary for existing studies showing changes in functional brain connectivity induced by cognitive training interventions is given in Table 3.

**Table 3:** **Neuroimaging studies showing changes in brain FC induced by cognitive interventions**.

Study	Modality	Training task	Control group	Population	Training period
Strenziok et al. (2014)	fMRI, DTI	Rise of Nations, Brain Fitness, Space Fortress	-	OA	6 weeks
Jolles et al. (2013)	fMRI	a verbal WM task	-	YA and CH	6 weeks
Takeuchi et al. (2013)	fMRI, ASL	WMT program	no training	YA	27 days
Langer et al. (2013)	EEG	Tatool (adaptive WM training)	tasks with low WM demand	YA	4 weeks
Heitger et al. (2012)	fMRI	a motor learning task	-	YA	4 days
Voss et al. (2012)	fMRI	Space Fortress	-	YA	20 h
Bassett et al. (2011)	fMRI	a motor learning task	-	YA (musical instruments)	5 days
Stevens et al. (2010)	fMRI	visual semantic classification tasks	-	YA	15 min
Albert et al. (2009)	fMRI	a motor learning task	a motor performance task	YA	11 min
Lewis et al. (2009)	fMRI	a shape-identification task	-	YA	2–9 days

*YA: young adults, OA: old adults, CH: children*.

## Conclusion

In this review article, we proposed that biomarkers based on brain connectome approach could be useful for a brain enhancement system by optimizing effectiveness of cognitive training interventions on learners’ learning process. For this purpose, we first introduced studies on cognitive training interventions. A number of studies have shown that the brain cognitive functions could be improved through cognitive training interventions such as WM training or video game training, and brain activities were modulated as a result of the training (Klingberg, 2010). Although such cognitive interventions are effective, their effectiveness can be different among individuals and they may even vary depending on mental state at the moment of the training. If the task that the learner is performing is too difficult or too demanding, it would be difficult for them to keep the motivation and concentration on the facing task and some of them can be exhausted from the excess of cognitive workload, resulting in a poor or a lack of improvement in cognitive functions. An adaptive assistance system for cognitive training through monitoring the learners’ mental states such as cognitive workload using objective biomarkers is a useful approach to facilitation and optimization of the learning progress. As introduced above, a number of biomarkers have been proposed for monitoring mental states including cognitive workloads. Most of the biomarkers are based on spectral properties of EEG signals or ERPs, and the extracted features are often subjected to cutting-edge mathematical tools based on machine learning theory to discriminate mental states (Kothe and Makeig, 2011). Additionally, some biomarkers can be used to predict individuals’ intelligence or learners’ subsequent learning rate (Langer et al., 2012). If optimal mental states for ongoing training can be replicated in the brain through the adjustment of the cognitive workload, neurofeedback, or other cognitive training scheme, we could optimize the effectiveness of the training process.

Furthermore, we have introduced the functional connectome approach, which is mainly based on the graph theory (Sporns, 2014). The brain is a complex network consisting of spatially distributed regions dedicated to different functions, and it is proposed that cognitive functions emerge from dynamic interactions of several brain areas, not a result of an activation of a single brain region (Bressler and Menon, 2010). As such, the number of publications regarding brain network is drastically increasing (Friston, 2011). We have proposed here that the brain connectome can be a useful approach not only for elucidating mechanisms underlying brain cognitive functions, but also for detection of mental states. The graph theoretical network metrics can be biomarkers of cognitive states, as shown in previous studies on cognitive workload (Ginestet and Simmons, 2011; Kitzbichler et al., 2011) or mental fatigue (Breckel et al., 2013; Sun et al., 2014a). Several studies have shown that the brain functional and structural network connectivity can be altered through cognitive interventions, and the graph theoretical network metrics have shown the reorganization of topological architecture of the brain functional network over multiple temporal scales (i.e., minutes, days, weeks). These results suggest that the functional connectome approach as well as conventional biomarkers would be effective methods for boosting learning progress of learners during the course of cognitive training.

## Conflict of Interest Statement

The authors declare that the research was conducted in the absence of any commercial or financial relationships that could be construed as a potential conflict of interest.

## Footnotes

^1^www.lumosity.com

^2^www.cognifit.com

^3^www.cogmed.com

^4^www.cogmed.com/program

^5^junglememory.com/
